# Risks and Management of Central Serous Chorioretinopathy in a Middle-Aged Female

**DOI:** 10.7759/cureus.51325

**Published:** 2023-12-30

**Authors:** Reshma Annam, Pravallika Padyala, Preethi Annam, Sailaja Nandennagari, Krupavaram Bethala

**Affiliations:** 1 Medicine, Windsor University School of Medicine, Saint Kitts, KNA; 2 Premedicine, Baylor University, Waco, USA; 3 Surgery, Avalon University School of Medicine, Curacao, CUW; 4 Medicine, Avalon University School of Medicine, Curacao, CUW; 5 Pharmacology and Therapeutics, Centre of Excellence in Pharmaceutical Sciences, School of Pharmacy, KPJ Healthcare University College, Kuala Lumpur, MYS

**Keywords:** photoreceptor cell dysfunction, subretinal fluid, retinal pigment epithelium (rpe), oct (optical coherence tomography), central serous chorioretinopathy

## Abstract

Central serous chorioretinopathy (CSC) is the buildup of fluid in the posterior pole distorting the vision resulting from either neurosensory or retinal pigment epithelial detachment. This is a case report of a 33-year-old female complaining of cloudiness in the left eye who was subsequently diagnosed with CSC using optical coherence tomography (OCT). Remission was observed in four months, possibly from ocular drop treatment or spontaneous. Our case report is unique as the incidence of CSC is more common in males, almost six times higher than in females. Also, the etiology of CSC in this case report is questionable because all the risks are excluded from our patient history.

## Introduction

Central serous chorioretinopathy (CSC) is a disease of fluid buildup in the posterior pole leading to vision distortion from either neurosensory or retinal pigment epithelial detachment in the macular region. The retinal pigment epithelium (RPE) is a layer of cells that sits between the retina and choroid. When excess fluid builds up under the retina, the RPE detaches and causes visual distortions such as blurred and dim vision. The patient may often experience micropsia, metamorphopsia, changes in color vision, alterations in contrast sensitivity, etc. [1]. CSC primarily impacts males between 30 and 50, who are more likely to develop CSC than women [2].

Approximately half of the patients diagnosed with CSC have at least one relative with disease findings identified during eye examination. In the USA, CSC has an incidence of about 9.9 per 100,000 men and 1.7 per 100,000 women, which is approximately six times less compared with men [3]. Although the incidence of CSC is greater in Asians compared with Caucasians, the reason may be due to seasonal and climatic variations, whose validation is required by future researchers [4]. Most patients recover spontaneously within three to four months; however, the recurrence rate can be as high as 20-50%.

## Case presentation

A 33-year-old female visited our clinic complaining of a 20-day history of cloudiness in the left eye on January 16, 2021. The patient denies any past medical history of diabetes mellitus, hypertension, or any other medical illnesses, and she claims she is very healthy and up to date with her annuals. Professionally, she is a teacher, so her vision is significantly causing inconvenience to her profession.

A slit lamp examination shows no evidence of inflammation in either eye. Table 1 lists the ocular examination.

**Table 1:** Measurements using optical coherence tomography of both eyes

Parameter	Right	Left
Visual acuity (corrected)	20/20	20/70
Intraocular pressure	16 mm of Hg	17 mm of Hg
Lens	Clear	Clear
Anterior segment	Normal	Normal
Fundus	Dilated, normal disc, macula and blood vessels	Round, shallow, well-delineated, neurosensorial macular detachment, no hemorrhages or exudates
ILM-RPE: Thickness central subfield (µm)	252	596
ILM-RPE: Volume cube (mm^3^)	11.2	13
ILM-RPE: Thickness average cube (µm)	311	361

**Figure 1:**
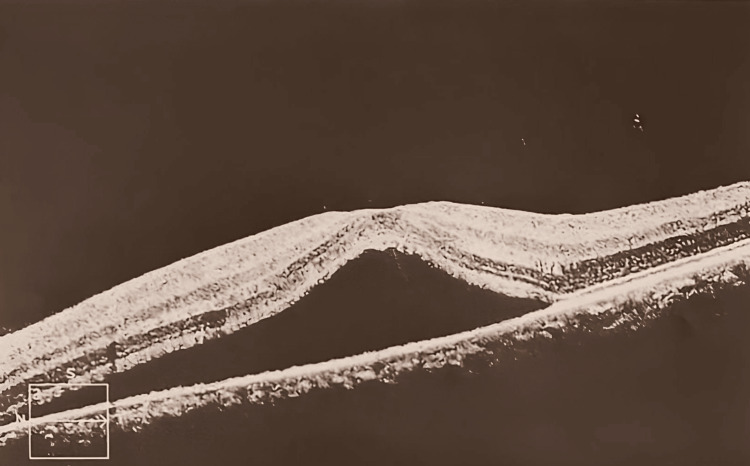
Chronic serous chorioretinopathy, left eye

Optical coherence tomography (OCT) was performed on January 18, 2021, and confirmed the diagnosis of central serous chorioretinopathy (Figure 1), thinking that stress may be the etiology as no supportive medical history was known from the patient. The patient was reassured and counseled on managing stress, as it is thought to be the principal cause of her condition. The patient has been prescribed ocular drops for the left eye, Lutein 20 mg PO, and vitamin E PO, expecting improved visual acuity and preventing complications such as RPE rip, irreversible visual decline, etc.

**Figure 2:**
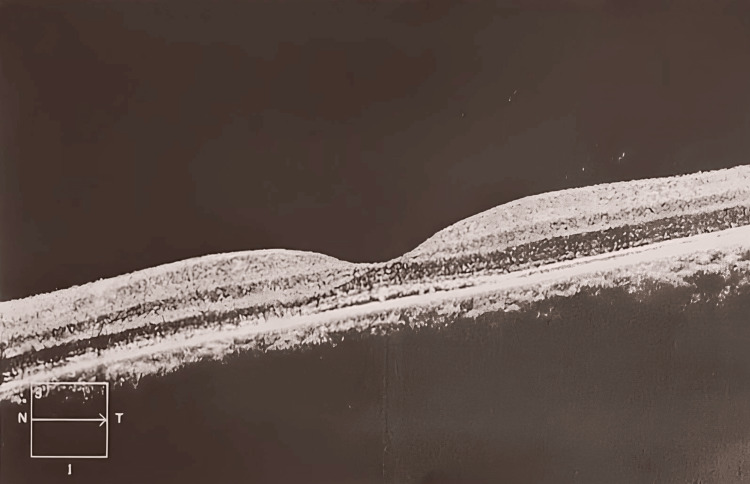
Chronic serous chorioretinopathy in remission, left eye

Fundoscopy and OCT were performed on the patient’s follow-up visit on May 21, 2021, which revealed serous retinal detachment in remission (Figure 2). The best corrected visual acuity at this visit was 20/20, and no further complications were noted.

## Discussion

Detachment of the serous retina, or retinal pigment epithelium, is the hallmark of CSC. Most patients with CSC are middle-aged or older, and the most commonly observed are males, which makes our case report as rare as our patient is female with an unknown cause of stress. Females, like our patients, rarely exhibit CSC. Although the condition often resolves on its own, it can occasionally worsen and result in irreversible damage to the retinal pigment epithelium and photoreceptors, which distorts the vision.

In a comparative study of gender variation in central serous chorioretinopathy by Hanumunthudu et al., female subjects had better visual acuity and outcomes with minimal diffuse RPE damage. This study also claims that ethnic variations are not responsible for the differences seen in the study [5].

Most common etiological factors, including pregnancy, higher testosterone levels, exogenous corticosteroid exposure, Helicobacter pylori infection, hypertension, insomnia, autoimmune disorders, and type A personality behaviors, have been independently linked to CSC. However, in our patient, none of these are noticed except that she is a competitive individual, which makes the cause a lack of sleep or idiopathic, of which we are not sure.

As per Bing Liu et al. in 2016, in a systematic review and meta-analysis, it was reported that in a total of 9839 patients from 17 studies, various risk factors were reported: hypertension (OR = 1.7; 95% CI: 1.28-2.25), steroid usage (OR = 4.29; 95% CI: 2.01-9.15), psychopharmacologic medication use (OR = 2.69; 95% CI: 1.63-4.45), insomnia (OR = 1.90; 95% CI: 1.28-1.83), autoimmune diseases (OR = 3.44; 95% CI: 1.90-6.26), type-A behavior (OR = 2.53; 95% CI: 1.08-5.96), and infections such as Helicobacter pylori (OR = 3.12; 95% CI: 1.81-5.40) [6].

Pharmacological therapy includes mineralocorticoid and glucocorticoid receptor oral antagonists, MR antagonists, specifically eplerenone, potassium channel blockers such as spironolactone, mifepristone, antioxidants, etc. Even though efficacy with Bevacizumab and Ranizumab is excellent in the first three months, sustainability beyond six months is questionable [7].

Non-pharmacological therapies such as LASER and photodynamic therapy are showing promising results as well, but prospective studies, controlled designs, and extensive sample studies still need to be included. Subretinal fluid (SRF) often settles on its own and runs its course naturally. However, chronic or recurrent SRF causes significant vision loss in certain instances due to RPE atrophy and photoreceptor cell dysfunction. Genome-wide association studies have been conducted for CSC, and several potential genes have been investigated. The complement factor H (CFH) gene is known to have variations related to CSC in several ethnicities.

As said above, acute CSC has a relatively good prognosis compared to chronic CSC, which may lead to loss of visual acuity to some extent. However, spontaneous remission is very popularly observed with acute CSC, leading to loss of retinal detachment and regaining visual acuity.

## Conclusions

In our case report, treatment for CSC included ocular drops, Lutein 20 mg, and vitamin E PO given to the patient. Five months post-treatment, improvement in visual acuity and subretinal fluid resolution may be the effects of treatment or natural spontaneous disease remission. Also, we are not sure whether there will be a recurrence of the CSC in the patient as it is prevalent, and the recurrence can be treated with the same as the above prescription, either by keeping the same or increasing the dose or changing to another medication.
